# An Unusual Cause of Fetal Stroke: Secondary HSV Infection

**DOI:** 10.1155/2019/7604076

**Published:** 2019-08-29

**Authors:** Tara Banaszek Daming, Sherri Jackson

**Affiliations:** University of Missouri, Kansas City School of Medicine, Saint Luke's Hospital of Kansas City, USA

## Abstract

Herpes simplex virus (HSV) is a recognized cause of neonatal infection, with hematologic vertical spread usually only during a primary infection. This is an unusual case with hematologic spread of an HSV 2 infection resulting in a fetal stroke, not associated with a primary outbreak during pregnancy. A 25-year-old multigravida at 26 weeks was diagnosed with a large fetal stroke. Previous ultrasounds showed normal anatomy. She had preterm labor and delivery, with unsuccessful neonatal resuscitation. Vesicular lesions were noted on the infant. Both the lesions and the cerebral spinal fluid were positive for HSV 2. She had documented HSV infection prior to pregnancy, indicating that this was due to a hematologic secondary infection, as there was no rupture of membranes or evidence of other modes of transmission. This case shows that, while unusual, HSV hematologic vertical transmission can occur in both primary and secondary infection during pregnancy. Infection screening is worthwhile in unexplained fetal strokes.

## 1. Introduction

Herpes simplex virus (HSV) is a recognized cause of perinatal infection, with hematologic vertical transmission only previously reported during a primary infection. Transmission risk from secondary HSV is primarily due to cervicovaginal transmission during birth [1]. This case outlines a secondary HSV infection that lead to hematologic vertical transmission in the second trimester resulting in a fetal stroke.

## 2. Case Report

**Figure 1:**
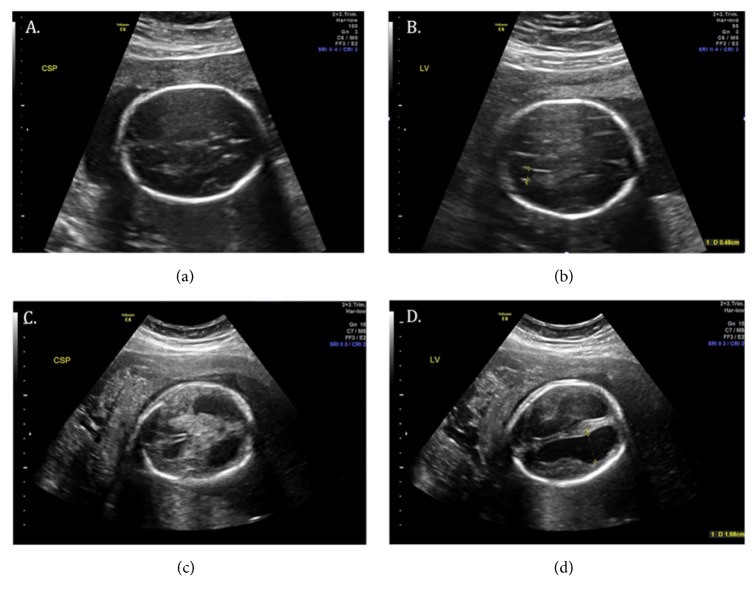
Normal brain anatomy seen at 23 weeks gestation (a, b) and massive ventriculomegaly and distortion seen at 26 weeks gestation (c, d). CSP= cavum septum pellucidum; LV= lateral ventricle.

**Figure 2:**
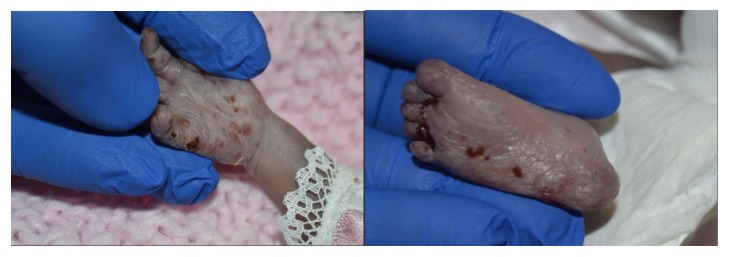
Neonatal vesicles present at birth. A swab was taken of the hand which confirmed HSV2 by PCR.

This is a case of 25-year-old G4P1021 diagnosed with an in utero fetal stroke at 26 weeks gestation. She had ultrasounds at 19 weeks and 23 weeks, which showed no anatomic abnormalities (see Figures 1(a) and 1(b)). She presented to labor and delivery at 26 weeks complaining of vaginal spotting. Labor and rupture of membranes were ruled out; however she had a Category II tracing with minimal to absent variability. Ultrasound exam revealed severe ventriculomegaly, a left-sided frontal porencephalic cyst, enlarged 4^th^ ventricle, mild abdominal ascites, open hands, and echogenic bowel (see Figures 1(c) and 1(d)). A thrombophilia panel, antiphospholipid panel, cytomegalovirus and toxoplasmosis antibodies, and neonatal alloimmune thrombocytopenia panel were ordered and were negative. She went into spontaneous preterm labor at 26 weeks 4 days' gestation and delivered a female infant, weighing 895g with APGARS of 1, 1, and 1, at 1, 5, and 10 minutes of life. Heart rate was in the 60s and abnormal rigid tone was noted. Intubation did not improve saturations (never over 60% despite correct placement confirmed), and chest x-ray showed bilateral whited out lungs. Compressions and chest tube placement did not yield improvement. Umbilical vein catheterization was unsuccessful and surfactant and epinephrine were given via the endotracheal tube. Despite extensive resuscitative efforts, there was no improvement in the clinical status, and the mother opted to switch to comfort care. Vesicular lesions were noted on the infant, which were positive for HSV2 by PCR (see Figure 2). A postmortum ventricular tap was performed and cerebral spinal fluid was also positive for HSV 2.

Her first clinically documented HSV outbreak was immediately prior to pregnancy and she was treated with acyclovir. She became pregnant approximately 5 weeks later. A 6-week ultrasound confirmed those dates. She was seen for her initial prenatal exam at 11 weeks, which confirmed a positive HSV-2 IgG. She had no further outbreaks during pregnancy, and her pregnancy was otherwise uncomplicated. She was not on suppression during her pregnancy, though it was planned for 36 weeks.

## 3. Discussion

Herpes simplex virus (HSV) has been a recognized cause of neonatal infection for decades, with the majority of cases occurring due to vaginal or cervical transmission [1]. Vertical hematologic transmission has universally been reported during primary infections, with complications including abortion, preterm labor, congenital malformations, and stillbirth [1, 2]. Secondary HSV infection has not been associated with those same risks [3]. Three-fourths of infections are due to cervicovaginal exposure, and congenital herpes typically presents as sepsis [4]. Congenital herpes is separated into three categories: skin, eyes, and mucosa (SEM), CNS-associated infection, and disseminated herpes [4]. CNS-associated infection manifests as developmental delay, epilepsy, blindness, and cognitive disabilities, without findings of prenatal stroke [4].

After review of the literature, no other fetal strokes were reported due to primary or secondary HSV vertical infection. HSV hematologic vertical transmission can occur in secondary infection, which was proven in this case by PCR analysis. Clinical manifestations of hematologic vertical transmission can include neonatal stroke, as seen in this case, and can present at gestational ages beyond the normal anatomic screening period. The majority of neonatal strokes have unknown causes, and, out of the known risk factors, the most common are alloimmune thrombocytopenia and trauma [5]. This case highlights that infection screening, including HSV, may have a role in prenatal stroke work-up when other etiologies are ruled out.
